# Endoscopic Management Using Novel Haemostatic Agents for Immediate Bleeding during Endoscopic Retrograde Cholangio-Pancreatography

**DOI:** 10.1155/2023/5212580

**Published:** 2023-04-10

**Authors:** Cosmas Rinaldi Adithya Lesmana, Sharon Sandra, Maria Satya Paramitha, Rino Alvani Gani, Laurentius A. Lesmana

**Affiliations:** ^1^Department of Internal Medicine, Hepatobiliary Division, Dr. Cipto Mangunkusumo National General Hospital, Medical Faculty Universitas Indonesia, Jakarta, Indonesia; ^2^Digestive Disease & GI Oncology Center, Medistra Hospital, Jakarta, Indonesia; ^3^Gastrointestinal Cancer Center, MRCCC Siloam Semanggi Hospital, Jakarta, Indonesia

## Abstract

Bleeding after endoscopic sphincterotomy (ES) remains as a major challenge during ERCP procedure. Standard endoscopic haemostatic procedures have demonstrated good performance for bleeding control. Novel endoscopic haemostatic agents have also been widely used in gastrointestinal bleeding management. Regardless, there is still a paucity of high-quality evidence evaluating the practicality of these agents in ERCP. This case series study was performed on the patients who underwent ERCP procedure in a tertiary referral private hospital within 2 years period. Post-ES immediate bleeding is defined as the onset of bleeding at the time of sphincterotomy. Treatment groups for post-ES bleeding are divided into (1) standard haemostatic methods and (2) novel haemostatic agents. There were 40 patients who received standard haemostatic treatment and 60 patients who received novel haemostatic agents. Initial haemostasis was achieved in all patients. Two patients who received standard haemostatic treatment had rebleeding. Meanwhile, no patients in novel haemostatic treatment group had rebleeding. In conclusion, novel haemostatic agent can be considered as an easy and practical method in daily practice, especially when an ERCP procedure is performed. Further studies with larger sample size which, if possible, can also include a cost-effectiveness analysis are still required to implement these agents as a standard procedure in clinical practice. (This abstract has been presented at the American College of Gastroenterology meeting October 2021).

## 1. Introduction

Endoscopic sphincterotomy (ES) is the cornerstone of therapeutic endoscopic retrograde cholangiopancreatography (ERCP) for various pancreaticobiliary disorders. Nevertheless, bleeding is still considered as one of the major complications after pancreatitis. Immediate bleeding during therapeutic ERCP might occur in 7.4%–12.1% of patients undergoing ES procedure [1, 2], whereas delayed bleeding after ERCP procedure can be observed in 2% of patients [3, 4]. Immediate bleeding usually tends to be self-limiting or can be easily treated endoscopically [5].

Generally, the clinical approach to post-ES bleeding largely depends on the degree of bleeding and whether the bleeding episodes can be treated with conservative therapy and/or endoscopically. Aside from the timing of bleeding (immediate or delayed), clinical manifestation of the bleeding also defines the severity of post-ES bleeding. Successful management, however, can be quite challenging due to anatomy of the papilla, bleeding sites, and the side-view duodenoscope maneuver. Another way to manage post-ES bleeding is by administering blood transfusion and/or angiographic intervention or even surgical approaches [2].

**Figure 1:**
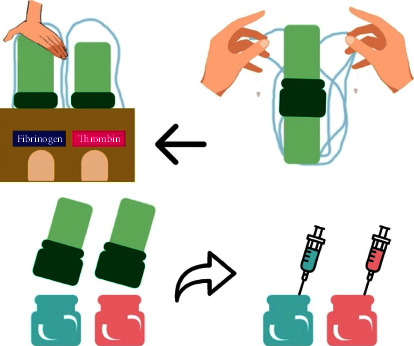
Beriplast solution preparation before injection procedure.

Standard endoscopic haemostatic methods, such as epinephrine injection, thermal coagulation, and mechanical approaches (balloon compression and hemoclip application), have been studied in the past. All of these methods, which can be performed alone or in combination, have been proven to lower post-ES bleeding morbidity and mortality as an initial modality [2]. Novel endoscopic haemostatic agents have also been used in gastrointestinal bleeding management, such as self-assembling peptide haemostatic gel, Purastat® (3-D Matrix Ltd, Tokyo), and fibrin sealant, Beriplast® (Aventis Behring Ltd., Marburg, Germany) (Figure 1). Purastat® will stick to and seal ruptured blood vessel. Moreover, it is also beneficial to induce haemostatic mechanical barrier [6]. On the other hand, Beriplast® mimics the final step of blood coagulation process, which in turn will produce fibrin clot [7, 8]. These novel haemostatic agents are easy to use with high applicability. Unfortunately, the number of studies discussing the utilization of these novel haemostatic agents during ERCP procedure is still lacking.

Therefore, this study aims to review the utilization of these novel haemostatic agents, especially in practical use, despite the application of standard haemostatic methods for immediate bleeding management during ERCP procedure based on tertiary referral center's clinical experiences.

## 2. Materials and Methods

This study was conducted using endoscopy database of patients who underwent ERCP procedure in our endoscopic unit, which was located in a tertiary referral private hospital within 2 years period. All ERCP procedures were performed by experienced endoscopists with more than 10 years of clinical experiences. In this study, therapeutic ERCP was defined as therapeutic procedure performed through an ERCP scope, including sphincterotomy, precut, or drainage. Patients who consumed anticoagulant or antiplatelet therapy or patients who had history of liver cirrhosis, chronic kidney disease, or other coagulation disorders were excluded in this study.

The degree of severity of post-ES bleeding in this study is determined based on the previous studies from Cotton et al. [9, 10]. Post-ES immediate bleeding is defined based on the onset of bleeding at the time of sphincterotomy, the requirement of blood transfusion, and the requirement of angiographic and/or surgical intervention. Based on the onset of bleeding, immediate bleeding is defined as bleeding which happens during or directly after the ES has been conducted. Delayed bleeding is defined as bleeding which occurs after several minutes of or longer after the ES has been conducted. Mild bleeding is defined as the presence of clinical and/or endoscopic evidence of oozing bleeding accompanied with a hemoglobin reduction of less than 3 g/dL without any requirement of blood transfusion. Meanwhile, moderate bleeding is defined as the presence of clinical and/or endoscopic evidence of large volume bleeding which require blood transfusion of ≤4 units, or which requires combined endoscopic therapy to achieve haemostasis without any need of performing angiographic and/or surgical intervention. Finally, severe bleeding is defined as the presence of clinical and/or endoscopic evidence of bleeding which requires more than 4 units of blood transfusion, or which needs to be treated with angiographic and/or surgical intervention.

**Figure 2:**
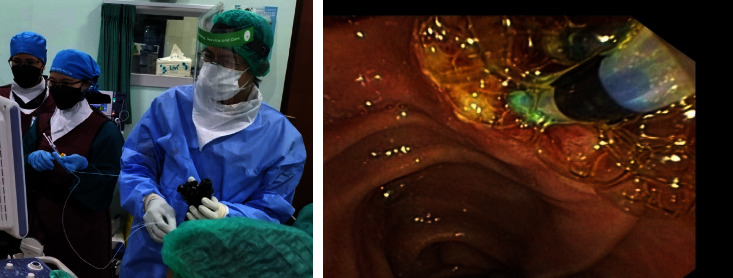
Beriplast injection procedure using two 5-Fr catheter.

**Figure 3:**
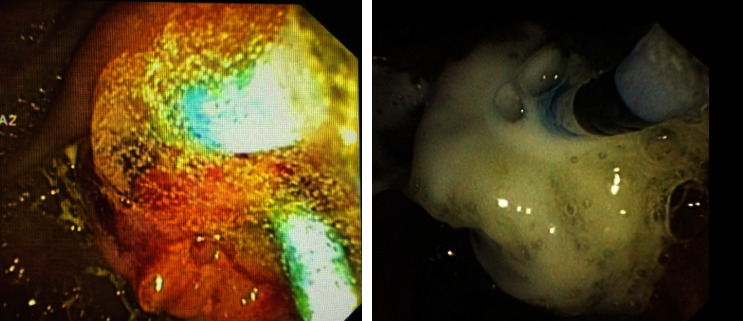
Images of purastat and beriplast injection to the papilla vater area.

Treatment methods for post-ES bleeding are divided into (1) standard haemostatic methods: balloon compression, adrenalin spooling, adrenalin injection, and submucosal contrast injection; and (2) Novel haemostatic agents: Beriplast® or Purastat®. Beriplast® is applied through two 5-FR ERCP cannula catheter inserted (side by side) through duodenoscope channel, whereas Purastat® is applied using 5.5-FR triple lumen ERCP cannula catheter inserted through duodenoscope channel (Figures 2 and 3).

Descriptive statistical analysis for continuous variables were calculated and reported as mean standard deviation or median (interquartile range) based on distribution of normality. Comparison numerical analysis between two groups was done by using independent t-test or Mann-Whitney test. Categorical variables were described using frequency distributions and were reported as *n*(%). Comparison categorical analysis between two groups was done using Chi-Square test. Statistical analyses were performed using Statistical Package for the Social Science (SPSS) for Windows, version 16.0. This retrospective database study has been approved by the ethics committee/institutional review board (IRB) of Medistra Hospital.

## 3. Results

Out of 392 patients who underwent ERCP with ES procedure, there were 304 patients who were included for our analysis. Characteristics of the patients are described in Table 1. Majority of the patients were males aged between 20–98 years old. The most common indication for ERCP procedure was choledocholithiasis (68%). From 304 patients, 67.1% of them did not experience any bleeding, while 27.3% experienced bleeding, in which most of them were suitable for immediate bleeding criteria (98%). Around 83% of the bleeding were classified as mild bleeding, while 17% of the bleeding events were considered as moderate bleeding. None of the patients experienced severe bleeding, which require other interventional procedures. Additionally, no delayed bleeding events were observed throughout the post-ERCPfollow-up. Among all of the patients who experienced immediate bleeding during the procedure, 40 patients received standard haemostatic treatment and 60 patients received novel haemostatic agents. Initial haemostatic was achieved in all patients. However, 2 patients who received standard haemostatic treatment had rebleeding and the bleeding was managed with novel haemostatic agent in one patient and argon plasma coagulation (APC) in another patient. No patients in the novel haemostatic treatment experienced rebleeding.

According to the degree of bleeding, 27% of the patients experienced mild bleeding, while only 2.3% of the patients experienced moderate bleeding. None of the patients experienced severe bleeding which require other intervention methods. From all patients who experienced mild bleeding, 34 patients initially received standard haemostatic treatment, while 9 patients initially received novel haemostatic treatment. None of the patients who received initial novel haemostatic treatment experienced moderate to severe bleeding. Additional study analysis revealed that increase of pancreatic amylase and lipase enzymes occurred more frequently in novel haemostatic treatment when compared to standard haemostatic treatment, but it was not statistically significant (*p*=0.086).

A subgroup analysis was conducted in this study (Table 2), showing that increased level of pancreatic enzymes was found more often in groups treated with monotherapy of novel agent treatment compared to groups treated with combination of conventional and novel agents (conventional vs. monotherapy novel: 7.5% vs. 25.6%; *p* 0.057 and conventional vs. combination of conventional and novel agents: 7.5% vs. 5.9%; *p* 1.000). A statistically significant difference in proportion of malignancy was also demonstrated by our subgroup analysis (conventional vs. monotherapy novel: 5% vs. 23.3%; *p* 0.040 and conventional vs. combination of conventional and novel agents: 5% vs. 11.8%; *p* 0.728). There was also a statistically significant difference in the median of age of the subjects involved in this study (conventional vs. monotherapy novel: 49 vs. 60 years; *p* 0.038 and conventional vs. monotherapy novel: 49 vs. 56 years; *p* 0.453).

## 4. Discussion

As mentioned in the introduction, this case series study was mainly conducted to evaluate the efficacy and practical applicability of the novel haemostatic agents despite the standard conventional methods. Fibrin sealant (fibrin adhesive or fibrin glue) agents themselves have been widely known as a blend of materials involved in the coagulation cascades, especially the blood clotting step. In general, fibrin sealants mainly have a role in the final steps of coagulation cascades. After platelets are deposited and aggregated, prothrombin will be cleaved into two fragments, including thrombin. Thrombin will then cleave the chains of fibrinogen into fibrin monomers. Polymerization of these monomers will form an insoluble fibrin. Afterwards, a clot, which consists of fibrin, adhesive glycoproteins, collagen, as well as plasma and cellular glycoproteins, will be attached to the site of injury. Aside from factor XIII (1–80 IU/ml) and thrombin (200–500 IU/ml), higher amount of fibrinogen is also included in the composition of almost all fibrin sealants [12].

In this study, the fibrin sealants used are two commercially available products. The first haemostatic agent (Beriplast®) contains human fibrinogen, human factor XIII, human thrombin, bovine aprotinin, and calcium chloride [12]. Meanwhile, the second agent (Purastat®) is a biocompatible synthetic peptide gel, consisting of a self-assembly of repeating sequence of amino acids, which will automatically form a three-dimensional nano fiber hydrogel scaffold once it directly comes in contact with blood [12]. These novel haemostatic agents have been used in the common gastrointestinal endoscopy procedures as well as in surgical procedures [13–16]; however, studies in endoscopic gastrointestinal bleeding management, especially in ERCP procedure using novel haemostatic agents are still lacking, and most of the studies are only based on case report, case series study, and even in small number of patients [8, 15, 17, 18].

In our study, there were only two patients who had rebleeding after standard endoscopic haemostatic management; while, in the novel haemostatic management group, there were no evidence of rebleeding (within 24 hours after procedure as well as one week afterwards). The most common standard haemostatic management during ERCP procedure are epinephrine injection and haemostatic clip. Both of them have been proven to be effective for managing immediate bleeding after ES procedure. Nonetheless, anatomical position of papilla, difficulty to push the injection needle out from the sheath, the rotation of hemoclip due to acute angulation directed by duodenoscope elevator, and risk of bleeding at the injection site can sometimes lead to possible morbid complications. Other potential side effects, such as pancreatitis and cholangitis, also need to be considered after these procedures [15, 19, 20]. Another option for treatment, such as endoscopic balloon compression at the bleeding site of the papilla is also a common procedure as it is easier to perform. Unfortunately, the evidence of its efficacy is still insufficient. In addition, the technical applicability also highly depends on how extensive the sphincterotomy procedure can be performed [20]. The balloon dilatation method for large or difficult common bile duct (CBD) stones extraction may also elevate the risk of pancreatitis, bleeding, and perforation [21]. This procedure also highly confides in the experiences of the operators since the guidewire location should be fixated inside the bile duct. Otherwise, it also may lead to harmful adverse events due to the difficulty in determining the anatomical location of bleeding. Some combination approach might also be needed for severe post-ES bleeding. Argon plasma coagulation (APC) is a thermal-based method which can be easily performed since no mucosal attachment is required. However, since the energy cannot be delivered directly into a specific bleeding area, careful approach must be conducted to avoid the pancreatic orifice, which may also increase the risk of pancreatitis [2, 16].

From our findings, the use of novel haemostatic treatment was associated with higher risk of elevated pancreatic enzymes, although the result was not statistically significant, in comparison to standard conventional methods. On the contrary, previous study showed that higher number of complications of pancreatitis was observed in the use of standard conventional methods, particularly epinephrine injection and thermocoagulation [10]. The risk of pancreatitis can also be increased due to this procedure [16]. A systematic review in 2019 showed that young age, female gender, dysfunction of sphincter of Oddi, history of previous post-ERCP pancreatitis can also be contributing factors towards PEP events. Papillary trauma due to difficulties in cannulation of CBD can also increase the incidence of PEP. Therefore, minimizing patient-related risk factors of PEP through careful patient selection can be a significant strategy to reduce the possibility of elevated pancreatic enzymes after ERCP procedure [22].

In this study, two novel haemostatic agents have been demonstrated as adequate and practical options for bleeding management after ES procedure. PuraStat® is a synthetic haemostatic material in prefilled syringe form. It is formulated from three types of amino acids that bound together to form peptide. When Purastat comes in contact with blood, the peptide-self assemble to form three-dimensional nanofiber scaffold which mimics human extracellular matrix. This matrix cause adhesive effect and closes the ruptured vessel which resulted in haemostatic control via mechanical barrier [6]. In the management of gastrointestinal bleeding, PuraStat has been demonstrated to achieve high initial haemostasis success rate (94%), as well as high secondary haemostasis success rate without any rebleeding after 3 (91%) and 7 days (87%). This study, however, was performed in adult populations with acute upper or lower active gastrointestinal bleeding according to Forrest classification [13]. To our knowledge, there has only been one case series which specifically evaluated the efficacy and applicability of Purastat® as the primary haemostatic agent in adult populations with ES-induced bleeding. Although the case series demonstrated Purastat as a potential agent to achieve initial heamostasis without any recurrent bleeding, there were only six subjects included in the study with relatively older age of the subjects (range of age: 72–88 years old). In the case series, similar with our study, one subject was also shown to have increased pancreatic enzyme after the procedure [23]. The transparent gel of Purastat can also tackle the challenge in distinguishing location of bleeding since it helps to visualize the area of bleeding better compared to the previous haemostatic agent (Hemospray®, Cook Medical, USA). We have also exhibited the possibility of utilizing two 5-Fr cannula catheters in the absence of double-lumen catheter, since the process needs the two-vial compound injected together. Another advantage of using these novel haemostatic agents is the smaller number of clogged catheter events which sometimes may occur in the application of Hemospray®. Even though the standard conventional haemostatic management is still quite effective in post-ES bleeding, however, the innovation method has made easier to perform for experience as well as less experience endoscopists.

There are several limitations of this study. Firstly, it was not designed as a randomized or head-to-head study. The retrospective study design might contribute to selection bias in the method. Nevertheless, this study is not aimed to evaluate the efficacy of new haemostatic agents since many convincing evidence had been gathered in the past about their effectiveness in other gastrointestinal bleeding management. Secondly, we did not perform any statistical analysis to evaluate the attributing factors of bleeding, which may become a recommendation for future studies. Several risk factors which can be evaluated are coagulopathy, the use of anticoagulation within several days of ES, the presence of cholangitis prior to ERCP, the presence of cirrhosis, dilated CBD, periampullary diverticulum, or CBD stones [2].

Overall, despite the previously mentioned limitations, to our knowledge, this is the first study which highlighted the practical applicability of novel haemostatic management in populations with post-ES bleeding. In comparison with standard conventional method, both novel haemostatic agents demonstrated more practical utilization, including the possibility of their application in the absence of less experienced endoscopists. This study showed the convenience of using novel haemostatic agents by tackling the main challenges which often occur in the use of standard conventional methods, for instance localizing the area of bleeding and performing the interventions through a side-viewing endoscope [10]. Future studies may further assess the superiority of these agents, in terms of cost-effectiveness, in randomized studies with larger samples. Analyzing their cost-effectiveness in the future can be a potential field of study since it is considered as a major issue in the application of novel haemostatic agents as the first choice for immediate bleeding management during ERCP procedures.

## 5. Conclusions

Novel haemostatic agents are easy and very practical in daily practice during therapeutic ERCP procedures. Further cost-effectiveness analysis with bigger data is needed to make this application as a standard procedure in clinical practice.

## Figures and Tables

**Table 1:** Characteristic comparison between conventional methods and novel haemostatic agents [11].

Variables	Total (*n* = 100)	Conventional methods (*n* = 40)	Novel haemostatic agents (*n* = 60)	*p* value
Sex				
Female (%)	41 (41%)	18 (45%)	23 (38.3%)	—
Male (%)	59 (59%)	22 (55%)	37 (61.7%)	0.648
Age (median, IQR)	56, 23	49, 22	59, 21	0.056
Laboratory data				
Hemoglobin (mean ± SD)	12.82 ± 1.58	12.92 ± 1.57	12.75 ± 1.59	0.626
Platelet count (mean ± SD)	305.4 ± 121.9	316.8 ± 138.39	297.78 ± 110.15	0.272
Prothrombin time (median, IQR)	13.1, 1.40	13.2, 1.53	13.05, 1.82	0.508
INR (median, IQR)	1.1, 1.56	1.1, 0.17	1.08, 0.14	0.913
Bilirubin (median, IQR)	4.7, 8.28	3.4, 8.44	5.23, 8.17	0.531
Etiology				
Nonmalignancy	86 (86%)	38 (95%)	48 (80%)	—
Malignancy	41 (41%)	2 (5%)	12 (20%)	0.068
Etiology				
Nonmalignancy				
Choledocholithiasis	68 (68%)	30 (75%)	38 (63.3%)	—
Cholelithiasis	6 (6%)	2 (5%)	4 (6.7%)	
Cholecystitis	1 (1%)	0 (0%)	1 (1.7%)	
Cholecystolithiasis	1 (1%)	0 (0%)	1 (1.7%)	
Mirizzi syndrome	3 (3%)	2 (5%)	1 (1.7%)	
Biliary stricture	6 (6%)	3 (7.5%)	3 (5%)	
Bile leak	1 (1%)	1 (2.5%)	0 (0%)	
Malignancy				
Klatskin tumor	4 (4%)	0 (0%)	4 (6.7%)	
Pancreatic tumor	4 (4%)	1 (2.5%)	3 (5%)	
Liver tumor	2 (2%)	1 (2.5%)	1 (1.7%)	
Cholangiocarcinoma	3 (3%)	0 (0%)	3 (5%)	
Ampullary tumor	1 (1%)	0 (0%)	1 (1.7%)	
Treatment method for bleeding				
Standard method				
Balloon compression	23 (23%)	23 (57.5%)	—	—
Epinephrine spray	2 (2%)	2 (5%)	—	
Epinephrine injection	4 (4%)	4 (10%)	—	
Submucosal contrast injection	11 (11%)	11 (27.5%)	—	
Novel haemostatic agents				
Purastat®	34 (34%)	—	34 (56.7%)	
Beriplast®	9 (9%)	—	9 (15%)	
Purastat® + balloon compression	7 (7%)	—	7 (11.7%)	
Beriplast® + balloon compression	10 (10%)	—	10 (16.6%)	
Timing bleeding				
None	2 (2%)	2 (5%)	0 (0%)	—
Immediate	98 (98%)	38 (95%)	60 (90%)	0.307
Delayed	0 (0%)	0 (0%)	0 (0%)	
Degree of bleeding				
Mild	83 (83%)	40 (100%)	43 (71.7%)	—
Moderate	17 (17%)	0 (0%)	17 (28.3%)	0.001
Severe	0 (0%)	0 (0%)	0 (0%)	
Rebleeding	2 (100%)	2 (100%)	0 (0%)	
Pancreatic enzyme levels				
Increased	15	3 (7.5%)	12 (20%)	
Not increased	85	37 (92.5%)	48 (80%)	0.086

**Table 2:** Subanalysis of monotherapy vs. combined therapy.

Variables	Conventional methods monotherapy (*n* = 40)	Novel haemostatic agents monotherapy (*n* = 43)	*p* value	Conventional methods monotherapy (*n* = 40)	Novel haemostatic agents combined (*n* = 17)	*p* value
Sex						
Female (%)	18 (45%)	16 (37.2%)	—	18 (45%)	7 (41.2%)	—
Male (%)	22 (55%)	27 (62.8%)	0.619	22 (55%)	10 (58.8%)	1.000
Age (median, IQR)	49, 22	60, 24	0.038	49, 22	56, 24	0.453
Laboratory data						
Hemoglobin (mean ± SD)	12.92 ± 1.57	12.91 ± 1.62	0.992	12.92 ± 1.57	12.37 ± 1.48	0.219
Platelet count (mean ± SD)	316.8 ± 138.4	308.8 ± 115.5	0.191	316.8 ± 138.4	269.9 ± 92.5	0.913
Prothrombin time (median, IQR)	13.2, 1.53	13, 2.0	0.541	13.2, 1.53	13.4, 2.20	0.637
INR (median, IQR)	1.1, 0.17	1.02, 0.11	0.571	1.1, 0.17	1.1, 0.12	0.420
Bilirubin (median, IQR)	3.4, 8.44	5.3, 9.01	0.466	3.4, 8.44	4.7, 5.77	0.875
Etiology						
Nonmalignancy	38 (95%)	33 (76.7%)	—	38 (95%)	15 (88.2%)	—
Malignancy	2 (5%)	10 (23.3%)	0.040	2 (5%)	2 (11.8%)	0.728
Etiology						
Nonmalignancy						
Choledocholithiasis	30 (75%)	27 (62.8%)	—	30 (75%)	11 (64.7%)	
Cholelithiasis	2 (5%)	2 (4.7%)		2 (5%)	2 (11.8%)	
Cholecystitis	0 (0%)	1 (2.3%)		0 (0%)	0 (0%)	
Cholecystolithiasis	0 (0%)	1 (2.3%)		0 (0%)	0 (0%)	
Mirrizi syndrome	2 (5%)	0 (0%)		2 (5%)	1 (5.9%)	
Biliary stricture	3 (7.5%)	2 (4.7%)		3 (7.5%)	1 (5.9%)	
Bile leak	1 (2.5%)	0 (0%)		1 (2.5%)	0 (0%)	
Malignancy						
Klatskin tumor	0 (0%)	4 (9.3%)		0 (0%)	0 (0%)	
Pancreatic tumor	1 (2.5%)	2 (4.7%)		1 (2.5%)	0 (0%)	
Liver tumor	1 (2.5%)	0 (0%)		1 (2.5%)	1 (5.9%)	
Cholangiocarcinoma	0 (0%)	2 (4.7%)		0 (0%)	1 (5.9%)	
Ampullary tumor	0 (0%)	1 (2.3%)		0 (0%)	0 (0%)	
Timing bleeding						
None	2 (5%)	0 (0%)	—	2 (5%)	0 (0%)	—
Immediate	38 (95%)	43 (100%)	0.442	38 (95%)	17 (100%)	0.879
Delayed	0 (0%)	0 (0%)		0 (0%)	0 (0%)	
Degree of bleeding						
Mild	40 (100%)	43 (100%)	—	40 (100%)	0 (0%)	<0.001
Moderate	0 (0%)	0 (0%)		0 (0%)	17 (100%)	
Severe	0 (0%)	0 (0%)		0 (0%)	0 (0%)	
Rebleeding	2 (100%)	0 (0%)	0.442	2 (100%)	0 (0%)	0.879
Pancreatic enzyme levels						
Increased	3 (7.5%)	11 (25.6%)	0.057	3 (7.5%)	1 (5.9%)	1.000
Not increased	37 (92.5%)	32 (74.4%)		37 (92.5%)	16 (94.1%)	

## Data Availability

All the supporting data are included in the manuscript. For any other requirements, please contact the corresponding author.
